# Novel Endothelial Cell-Specific AQP1 Knockout Mice Confirm the Crucial Role of Endothelial AQP1 in Ultrafiltration during Peritoneal Dialysis

**DOI:** 10.1371/journal.pone.0145513

**Published:** 2016-01-13

**Authors:** Wei Zhang, Marc Freichel, Frank van der Hoeven, Peter Paul Nawroth, Hugo Katus, Florian Kälble, Edgar Zitron, Vedat Schwenger

**Affiliations:** 1 Department of Nephrology, University of Heidelberg, Heidelberg, Germany; 2 Institute of Pharmacology, University of Heidelberg, Heidelberg, Germany; 3 Transgenic Service, German Cancer Research Centre, Heidelberg, Germany; 4 Department of Endocrinology and Metabolism, University of Heidelberg, Heidelberg, Germany; 5 German Center for Diabetes Research (DZD), Neuherberg, Germany; 6 Department of Cardiology, University of Heidelberg, Heidelberg, Germany; 7 Department of Nephrology, Klinikum Stuttgart, Stuttgart, Germany; French National Centre for Scientific Research, FRANCE

## Abstract

The water channel aquaporin-1 (AQP1) mediates about 50% ultrafiltration during a 2-hour hypertonic dwell in global AQP1 knockout (AQP1^-/-^) mice. Although AQP1 is widely expressed in various cell types including mesothelial cells, the ultrafiltration has been assumed to be mediated via endothelial AQP1 of the peritoneum. The partial embryonic lethality and reduced body weight in AQP1^-/-^ mice may reflect potential confounding phenotypic effects evoked by ubiquitous AQP1 deletion, which may interfere with functional analysis of endothelial AQP1. Using a Cre/loxP approach, we generated and characterised endothelial cell- and time-specific AQP1 knockout (AQP1^fl/fl^; Cdh5-Cre^+^) mice. Compared to controls, AQP1^fl/fl^; Cdh5-Cre^+^ mice showed no difference in an initial clinical and biological analysis at baseline, including body weight and survival. During a 1-hour 3.86% mini-peritoneal equilibration test (mini-PET), AQP1^fl/fl^; Cdh5-Cre^+^ mice exhibited strongly decreased indices for AQP1-related transcellular water transport (43.0% in net ultrafiltration, 93.0% in sodium sieving and 57.9% in free water transport) compared to controls. The transport rates for small solutes of urea and glucose were not significantly altered. Our data provide the first direct experimental evidence for the functional relevance of endothelial AQP1 to the fluid transport in peritoneal dialysis and thereby further validate essential predictions of the three-pore model of peritoneal transport.

## Introduction

Despite technical advances, peritoneal dialysis (PD) is still limited by peritoneal membrane (PM) changes resulting in ultrafiltration failure (UFF) [1]. According to the three-pore model of peritoneal transport, dysfunction of the water channel aquaporin 1 (AQP1) in peritoneal endothelial cells is a major cause of UFF [2]. It has been validated in global AQP1 knockout mice (AQP1^-/-^) that AQP1 mediated about 50% of ultrafiltration during a 2-hour hypertonic dwell [3].

AQP1 is the most abundant isoform in the highly vascularised peritoneal membrane (PM), and also the only one that has been consistently located in the capillary endothelium in various species including mouse [3, 4–9]. Hence, it has been assumed that the UF modification observed in AQP1^-/-^ mice is predominantly linked to AQP1 located in the endothelium of PM. To date, however, no experimental data have specifically pinpointed the role of endothelial AQP1 in the UF during PD.

Of note, AQP1 is broadly expressed in a variety of cell types and organs, including kidney, lung, mesothelial cells and red blood cells [10–14]. In particular, expression and function of peritoneal mesothelial AQP1 was regulated by buffer-dependent PD fluids [12]. Moreover, the global gene deficiency from early stages of development on may induce potential complex pleiotropic phenotypes and phenotypic consequences caused by compensatory mechanisms [15]. Indeed, the described AQP1^-/-^ mice exhibit systemic abnormalities, such as growth retardation with 10 to 15% reduced body weight and partial embryonic lethality with impaired survival *in utero* and/or in the early neonatal period [16, 17]. The reduced body weight in knockout mice was assumed to interfere with interpreting experimental outcomes, e.g. in studies of hypertension, drug and hormone metabolism, organ development, cell proliferation and apoptosis [18]. Body weight is also known to be an important parameter influencing peritoneal UF calculation [3].

In order to circumvent these limitations for the functional analysis of endothelial AQP1, we generated and characterised a novel endothelial cell (EC)-specific and time-specific inducible AQP1 knockout mouse model (AQP1^fl/fl^; Cdh5-Cre^+^) using a Cre/loxP system.

According to the three-pore model, during a hypertonic dwell both the transcellular transport of free water without solutes (FTW) across ultrasmall pores (AQP1) and the transport across small pores (UFSP) account for the total net UF (NUF) [2, 19–22]. In this study, we used the 1-hour mini-peritoneal equilibration test with 3.86% glucose (3.86% mini-PET) [23], which has been validated as being a simple, fast and more practical method for assessing peritoneal transport characteristics [23–25], to investigate the role of the endothelial AQP1 in fluid and solute transport across the PM.

## Results

Cre-loxP technology combined with inducible systems was used for generating a tamoxifen inducible endothelial cell-specific AQP1 knockout mouse model, which allowed for inactivating AQP1 expression in an inducible manner at any stage of mouse development.

### Generation and Characterisation of Floxed AQP1 Mice

**Fig 1:**
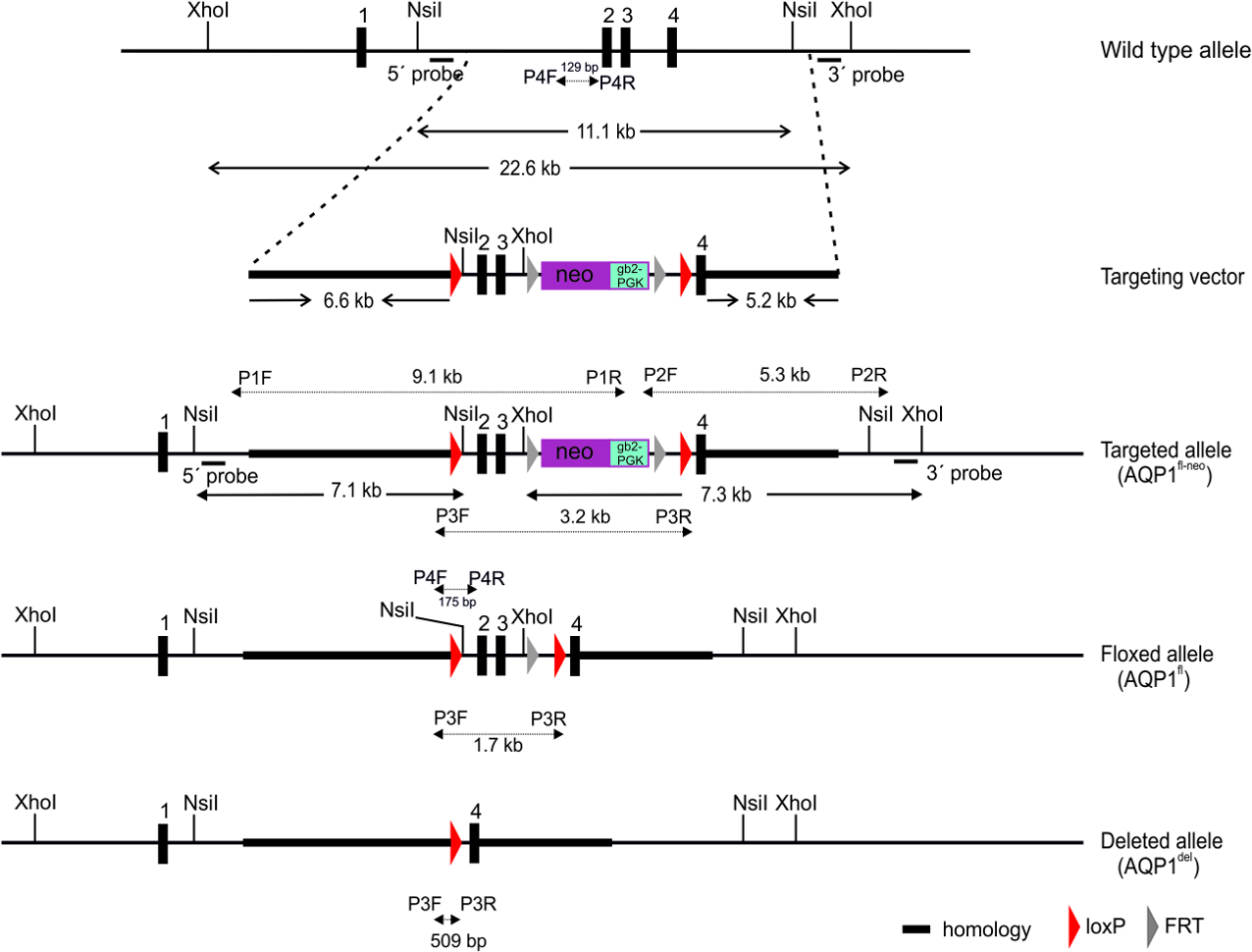
Targeting construct and screening strategies. A part of the wild type (+) allele of mouse AQP1 is shown with indicated exons (black boxes) and restriction enzyme sites. The targeting allele (fl-neo) is indicated with 3’ and 5’ targeting arms (thick lines), loxP / FRT sites and pro- and eukaryotic neomycin selection cassette (neo-gb2-PGK). The 5’ probe and 3’ probe for Southern blot located outside the targeting vector detect 11.1-kb (+) and or 7.1-kb (fl-neo) fragments from Nsi I-digested genomic DNA and 22.6-kb (+) and or 7.3-kb (fl-neo) fragments following Xho I-digestion genomic DNA, respectively. Mice carrying the floxed allele (AQP^fl-neo^) were crossed to FLPeR mice for excision of the FRT-flanked neo cassette. The resulting floxed mice (AQP^fl^) were crossed to Cdh5 (PAC)-CreERT2 (Cdh5-Cre) transgenic mice to excise exons 2 and 3 following tamoxifen induction, and then generate the AQP1 null allele (AQP1^del^) in endothelial cells. The P1F/1R, P2F/2R, P3F/3R and P4F/4R primers for PCR-based genotype analyses and the lengths of their responding PCR products are indicated.

To obtain a floxed AQP1 allele appropriate for Cre-mediated conditional inactivation of the AQP1 gene, a targeting vector was designed and constructed that could insert two loxP sites through homologous recombination flanking exons 2 and 3 of the AQP1 gene in mouse embryonic stem cells (ES cells) (Fig 1). These exons encode the crucial functional domains, the transmembrane domain (TMD) 4 and 5, as well as NPA motif (asparagine-proline-alanine) of loop E, which has been demonstrated to be implicated in the aqueous pathway [26].

**Fig 2:**
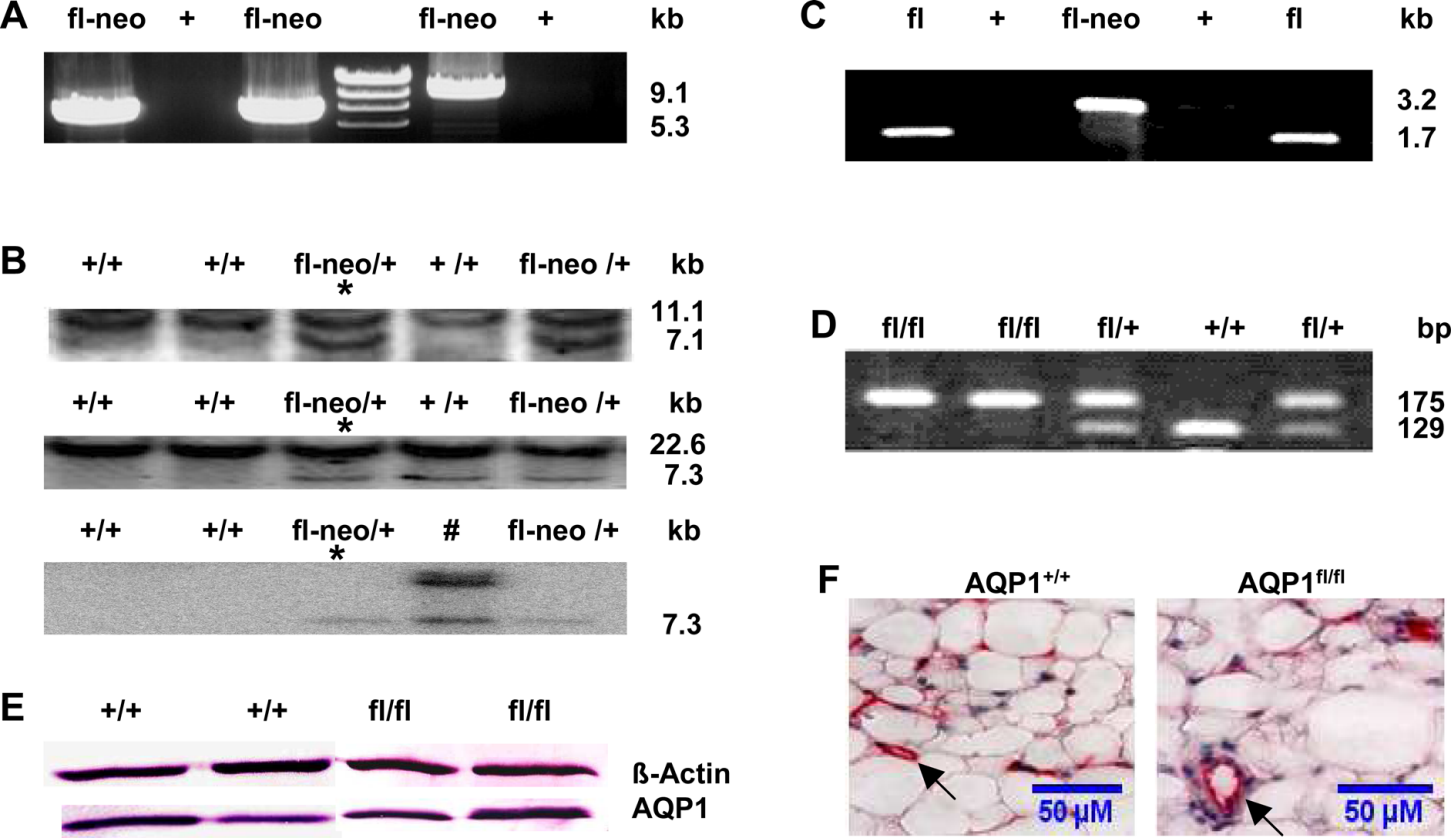
Generation and validation of the floxed AQP1 allele. (A) Both 5' and 3' homologous recombinants were screened by long-range PCR with neo-specific primers P1F/1R and P2F/2R. PCR of representative embryonic stem (ES) clones showed the targeted 5'-(9,1kb) and the 3'- (5.3kb) homologous recombination events, respectively. (B) Targeted ES clones in (A) were confirmed by Southern blot analysis. Using the 5' probe (upper panel), the 3' probe (middle panel), and the *neo* probe (lower panel), the representative resulting Southern hybridisation signals appeared upon digestion of genomic DNA from ES clones with the Xho I (3' probe and *neo* probe) or Nsi I (5' probe). The genotypes of WT (+) and targeted ES clones with neo cassette (fl-neo) and the size of the detected fragments are indicated. Detection of a single 7.3 kb fragment with the *neo* probe indicates a singular integration event, whereas one clone (#) showed an additional integration of the neo cassette. Germline transmission was obtained from the clone indicated with an asterisk following blastocyst injection. (C) Genotyping of AQP1^+/+^ (+), AQP1^fl-neo/+^ (fl-neo) and AQP1^flox/+^ (fl) mice by PCR using primers 3F and 3R. (D) WT (+/+), heterozygous (fl/+), homozygous (fl/fl) floxed alleles were distinguished by PCR with primers 4F and 4R. (E) Immunoblot of total protein fractions from visceral peritoneal (VP) homogenate probed with AQP1 antibody. Equal loading (40 μg of protein from each sample was verified using an anti-β-actin antibody. (F) AQP1 immunostaining showed normal localisation in the microvascular endothelium (arrows, stained in red) in VP and no apparent difference was observed between the AQP1^+/+^ and AQP1 ^fl/fl^ mice. Calibration bar: 50μM.

The correct genomic targeting of the AQP1 floxed construct was obtained and characterised as shown in two representative clones by long-range PCR (Fig 2A) and Southern blot (Fig 2B). Correctly targeted ES clones containing the AQP1^fl-neo^ allele were microinjected into C57BL/6 blastocysts to generate heterozygous AQP1^fl-neo^ mice. AQP1^fl-neo^ mice were crossed with FLPeR mice to remove the FRT-flanked neo cassette in their pups with an AQP1^fl/+^; FLPeR^+^ genotype (data not shown). AQP1^fl/+^; FLPeR^+^ mice were then backcrossed with C57BL/6 mice to generate AQP1^fl/+^ mice that were negative of both neo cassette and FLPeR. As shown in Fig 1 and Fig 2C, PCR analyses with primers P3F/3R distinguished AQP1^fl-neo^, AQP1^fl^ and AQP1^del^ allele, the latter of which was obtained by Cre-mediated deletion of exons 2 and 3. With this pair of primers, no PCR product was amplified from the wild type (WT) allele (+) because of the lack of sequences of primer 3R. Primers 4F and 4R were used to differentiate heterozygous floxed AQP1 mice (AQP1 ^fl/+^) from homozygous floxed AQP1 mice (AQP1^fl/fl^) (Fig 1 and Fig 2D).

Intercrosses of heterozygous AQP1^fl/+^ mice yielded 129 pups—33 wild-type (25.6%), 65 heterozygous (50.4%), and 31 homozygous (24.0%), which is according to the expected Mendelian ratio. Western blot (Fig 2E) and immunohistochemistry (IHC) analysis (Fig 2F) showed that the expression level and cellular distribution of the AQP1 gene in visceral peritoneum (VP) of AQP1^fl/fl^ mice were indistinguishable from those of AQP1^+/+^ mice. Survival, Mendelian inheritance of the AQP1^fl^ allele and unaltered expression of AQP1 proteins are all indications of normal gene expression from the AQP1 allele with loxP-flanked exons 2 and 3.

### Generation and Characterisation of an Endothelial Cell-specific AQP1 Knockout Mouse

Subsequently, AQP1^fl/fl^ mice were crossed with transgenic Cdh5 (PAC)-CreERT2 mice (Cdh5-Cre) carrying the Cre recombinase gene driven by the vascular endothelial cadherin Cdh5 promoter [27]. The AQP1^fl^ allele was converted to a AQP1 null allele (AQP1^del^) in a tamoxifen-inducible manner in early adult mice by EC-specific Cre-mediated excision of exons 2 and 3. In addition, AQP1^fl/fl^; Cdh5-Cre^+^ mice survived at a similar rate (1/53) as control AQP1^fl/fl^; Cdh5-Cre^-^ mice (0/57) during the period of 1–14 days following the last day of tamoxifen treatment until functional analyses.

**Fig 3:**
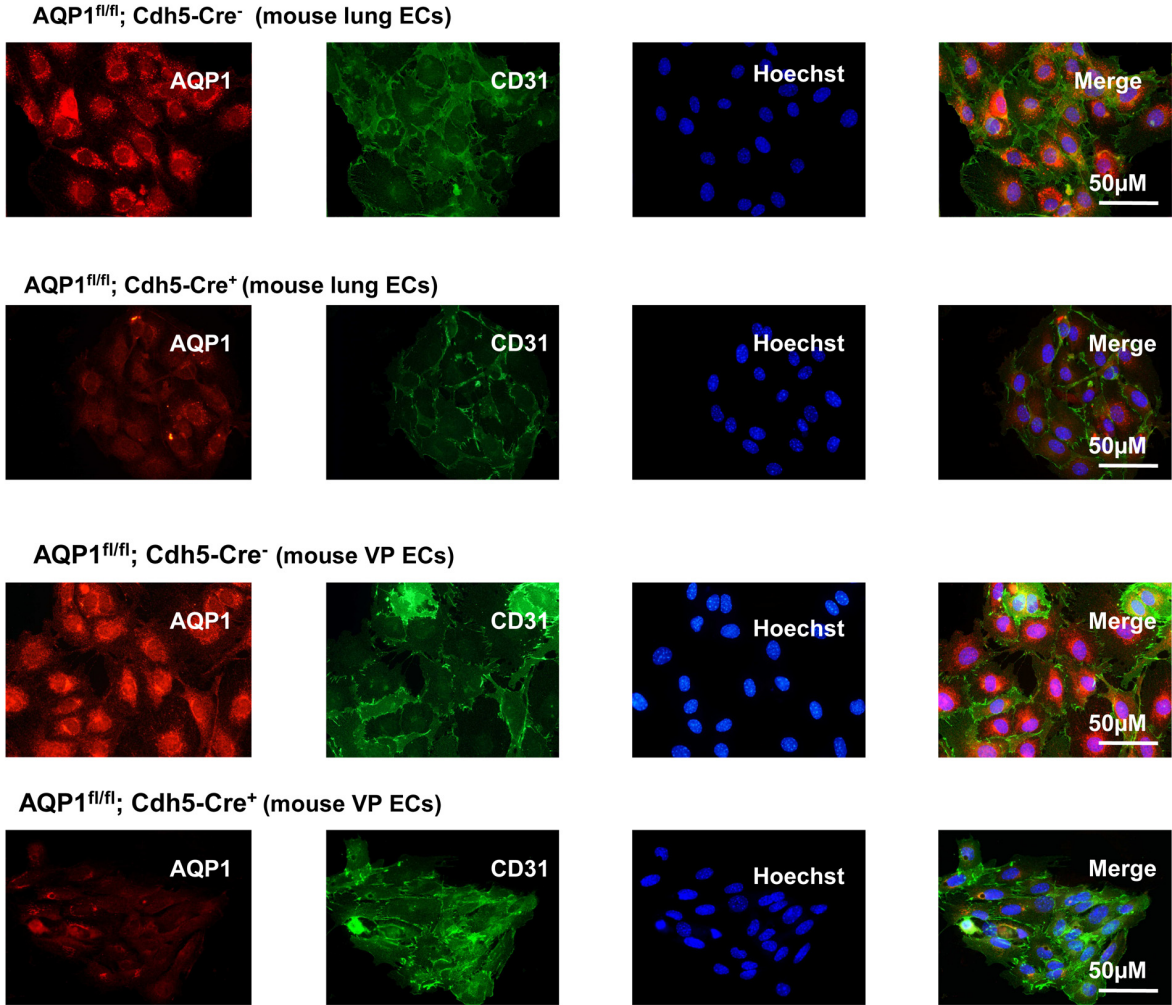
Detection of AQP1 expression in isolated endothelial cells from lung or visceral peritoneum (VP) by immunofluorescence. The representative isolated endothelial cells (ECs) from lung were immunostained for the EC marker CD31 (green, with Hoechst-stained blue nuclei) and AQP1 (red) in AQP1^fl/fl^; Cdh5-Cre^-^ and AQP1fl/fl; Cdh5-Cre^+^ mice (upper two panels). ECs showed a similar staining for the CD31, whereas ECs from AQP1^fl/fl^; Cdh5-Cre^+^ mice showed a markedly decreased staining for the AQP1 compared to those in AQP1^fl/fl^; Cdh5-Cre^-^ mouse. From the same mice, comparable results were observed in isolated ECs from visceral peritoneum (VP) (lower two panels). Calibration bar: 50μM.

**Fig 4:**
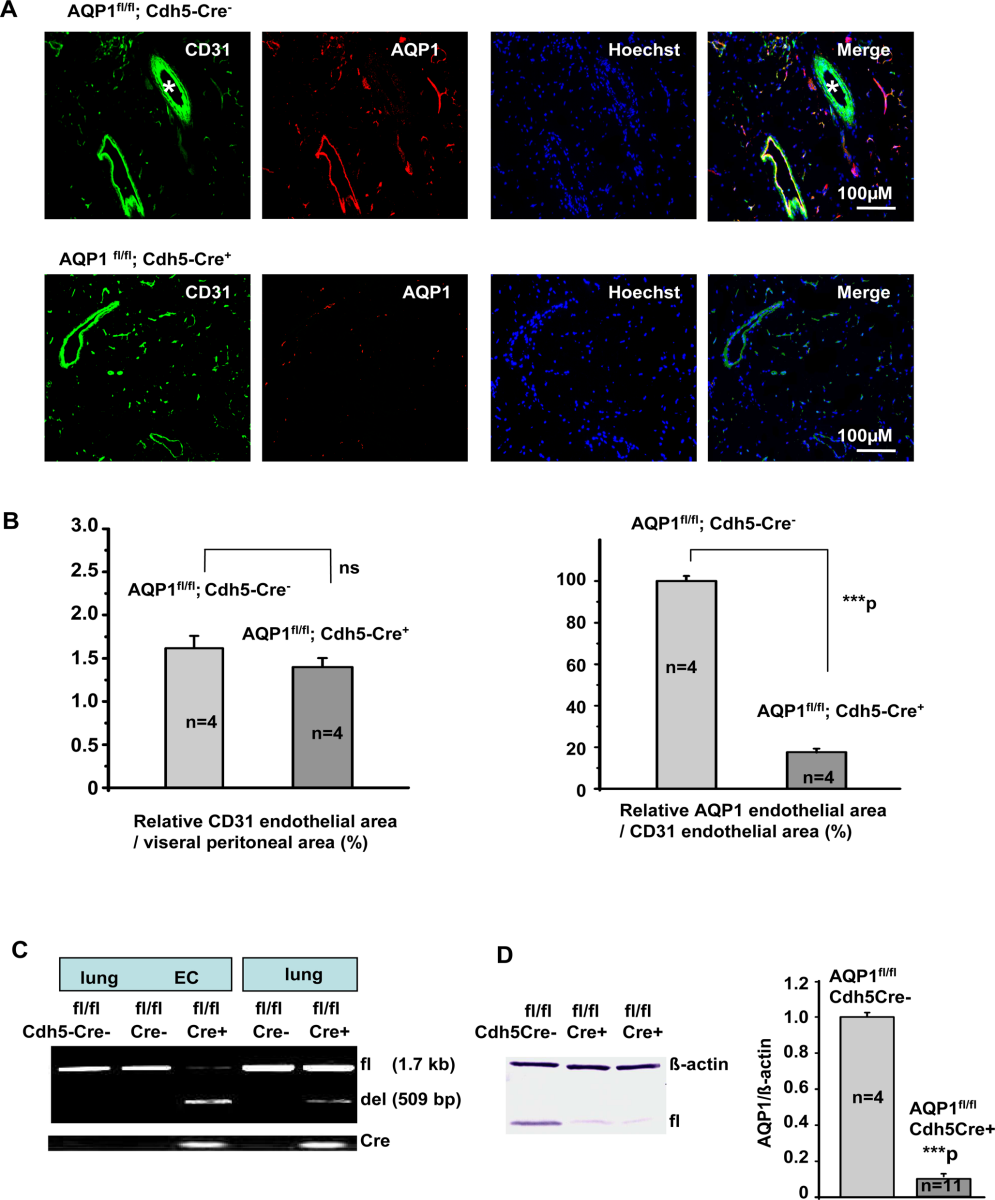
Characterisation of endothelial cell-specific AQP1 knockout mice. Both AQP1^fl/fl^; Cdh5-Cre^-^ and AQP1^fl/fl^; Cdh5-Cre^+^ mice were derived from AQP1^fl/fl^ and Cdh5-Cre breeding pairs. AQP1 null allele (del) were induced by tamoxifen. (A and B) Detection and quantification of AQP1 expression in the mouse visceral peritoneum by immunofluorescence. Immunoreactivity for AQP1 is located in the endothelium lining peritoneal capillaries and venules, but not in small arterioles (*). Sections from AQP1^fl/fl^; Cdh5-Cre^+^ mice showed similar CD31-labelled endothelial area as those from control AQP1^fl/fl^; Cdh5-Cre^-^ mice. In contrast, sections from AQP1^fl/fl^; Cdh5-Cre^+^ mice showed a nearly ~80.19% decrease in AQP1-labelled endothelial area compared to controls. ***P< 0.001 *versus* control group. Calibration bar: 100μM in (A). (C) PCR analysis of AQP1 null allele (del). DNA preparations from lung tissue and lung endothelial cells (ECs) were analysed. The floxed (fl) and null (del) alleles were distinguished by PCR with primer 3F and 3R. Cdh5-CreF/R primers were used to detect the Cre transgene. (D) Quantitative measurement of AQP1 gene expression in total protein fractions from lung ECs.

EC-specific Cre-mediated deletion efficiency was investigated by immunocytochemistry (ICC) staining of ECs isolated from lung or VP (Fig 3), and immunohistochemistry (IHC) staining of VP sections (Fig 4A and 4B). CD31-stained cells in both groups showed similar immunoactivity. In the absence of Cre recombinase, AQP1 was strongly expressed in the cultured ECs, but it was remarkably diminished in ECs with Cre recombinase expression. Quantification of the relative CD31-stained endothelial area in the VP sections showed no significant difference between the AQP1^fl/fl^; Cdh5-Cre^-^ mice and AQP1^fl/fl^; Cdh5-Cre^+^ mice (Fig 4B, left). This confirms that the endothelial area was not significantly altered by the deletion of AQP1 during development (3). Compared to control AQP1^fl/fl^; Cdh5-Cre^-^ mice, the endothelial AQP1 expression in VP of AQP1 ^fl/fl^; Cdh5-Cre^+^ mice was diminished by 80.2% (Fig 4B, right).

Representative Western blot (left) showed AQP1 protein expression in the lung ECs from control (AQP1^fl/fl^; Cdh5-Cre^-^) and endothelial AQP1-deficient mice (AQP1^fl/fl^; Cdh5-Cre^+^). Equal loading (10 μg of protein from each sample) was verified using an anti-β-actin antibody. Scanning densitometric analysis of AQP1 protein expression (AQP1 protein/β-actin) was done using Image J (right). Data are expressed as a ratio relative to the control group. Each bar indicates mean ± S.E. ***P < 0.001.

Comparison of the signal intensity obtained from lung and EC DNA samples extracted from the AQP1^fl/fl^; Cdh5-Cre^-^ and AQP1^fl/fl^; Cdh5-Cre^+^ mice indicates a high degree of specific excision of exon 2 and 3 in the ECs (Fig 4C).

Western blot analysis also demonstrated the efficiency of AQP1 deletion in lung ECs (Fig 4D). Compared to AQP1 ^fl/fl^ Cdh5-Cre^-^ mice (n = 4), the level of AQP1 protein was diminished in AQP1 ^fl/fl^ Cdh5-Cre^+^ mice (n = 11) by 89.0%.

### Clinical and Biological Parameters of the Endothelial Cell-specific AQP1 Knockout Mice

In comparison with control (AQP1fl/fl; Cdh5-Cre-) mice, AQP1fl/fl; Cdh5-Cre+ mice with the specific deletion of AQP1 in ECs showed no difference in an initial clinical and biological analysis at baseline including body weight (Table 1).

**Table 1:** Baseline clinical and biological parameters in the AQP1 mice

Groups	N	PNa (mmol/L)	Purea (mg/dL)	Pcreatinine (mg/dL)	Pglucose (mg/dL)	Osmolarity (mosmol/L)	BW (g)
**AQP1**^**fl/fl**^**;Cdh5-Cre**^**-**^	**6**	**140.08±1.42**	**45.16±3.63**	**0.19±0.02**	**349.17±63.45**	**329.83±4.36**	**21.59±1.22**
**AQP1**^**fl/fl**^**;Cdh5-Cre**^**+**^	**6**	**142.11±1.03**	**42.89±1.92**	**0.18±0.03**	**347.28±41.65**	**332.45±6.22**	**22.09±0.98**

BW, body weight; P, plasma concentration; all parameters were obtained from 8-week-old mice without processing 1-hour mini-peritoneal equilibration test (mini-PET) with 3.86% glucose.

### Role of AQP1 in UF and Small Solute Transport During Mini-PET

**Fig 5:**
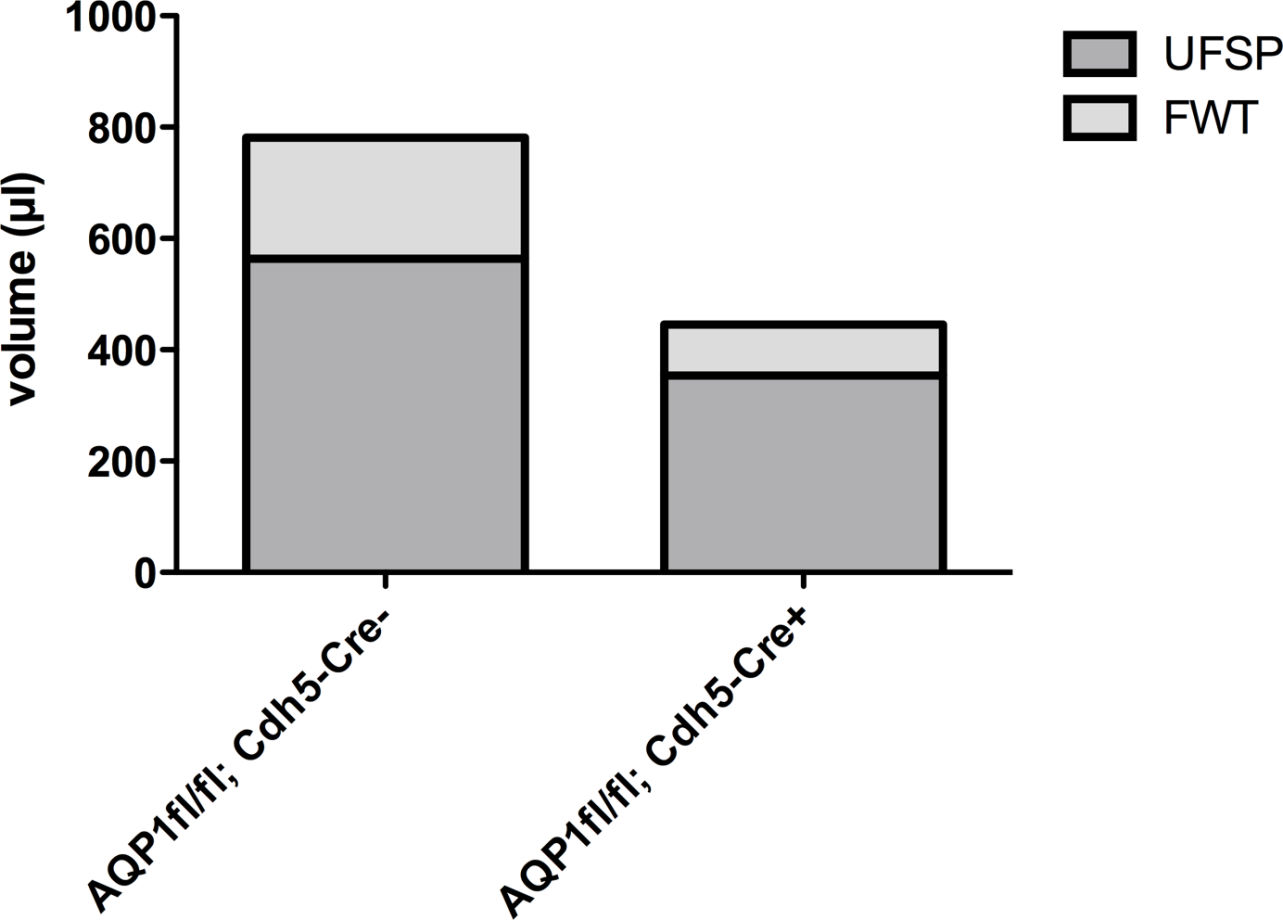
Ultrafiltration characteristics in the AQP1 mice. Net ultrafiltration (NUF) is the amount of UF through the small pores (UFSP) and free water transport (FWT). UFSP and FWT are highly hindered in AQP1 knockout mice (AQP1^fl/fl^; Cdh5-Cre^+^).

Table 2 and Fig 5 summarises the peritoneal transport characteristics during the 3.86% mini-PET. In control AQP1^fl/fl^; Cdh5-Cre^-^ mice, the parameters for transcellular water transport by AQP1 channels exhibited as a NUF of 780.83 ± 71.49 μl, considerable sodium sieving (8.71 ± 0.96%) and a FWT 217.07 ± 32.03 μl. In comparison with the control mice, the AQP1^fl/fl^; Cdh5-Cre^+^ mice showed a 43.0% decrease in NUF (445.00 ± 97.96 μl), 93.0% decrease in sodium sieving (0.61 ± 2.82%), and a 57.9% decrease in FWT (91.42 ± 48.23 μl). However, AQP1^fl/fl^; Cdh5-Cre^+^ mice demonstrated similar characteristics to control mice for small solute transport, such as urea and glucose.

**Table 2:** Water and small solute transport in the AQP1 mice.

Groups	AQP1^fl/fl^;Cdh5-Cre^-^ (N = 12)	AQP1^fl/fl^;Cdh5-Cre^+^ (N = 12)
BW (g)	23.84±0.82	23.14±0.94
NUF (μl)	780.83±71.49	445.00±97.96**
Na60D (mmol/L)	125.79±1.90	133.01±3.31^ns^
Na60P (mmol/L)	143.57±1.54	141.85±2.57
Na60D/P	0.88±0.01	0.94±0.03*
Sodium sieving (%)	8.71±0.96	0.61±2.82**
UFSP (μl)	563.76±75.43	353.58±75.34^ns^
FWT (μl)	217.07±32.03	91.42±48.23*
Glucose D60/D0	0.25±0.01	0.24±0.01^ns^
Urea 60 D/PBW (g)	0.81±0.01	0.85±0.02^ns^

NUF, total net ultrafiltration; BW, body weight; D, dialysate concentration; P, plasma concentration; V, effluent volume; 0 or 60, indicated dwell time (min); USFP, UF through small pores; FWT, free water transport

Data are expressed as mean±SE

*P < 0.05

**P ≤ 0.01 vs AQP1fl/flCdh5-Cre^-^

ns, not significant

## Discussion

Ultrafiltration (UF) is an important cause for peritoneal dialysis (PD) failure [1]. The three-pore model of peritoneal transport predicts that the water channel AQP1 is the ultrasmall pore located in the endothelial cells and mediates about 50% of ultrafiltration during a 2h-dwell. This concept has been validated experimentally in global AQP1 knockout mice (AQP1^-/-^) [6]. The present study bypassed the drawbacks of potential confounding phenotypic effects from ubiquitous deletion of AQP1 gene, using a well-characterised inducible endothelial cell-specific AQP1 knockout mouse model, to investigate the central functions of endothelial AQP1 in UF during PD.

To achieve cell-specific and /or conditional gene knockout, first we generated the AQP1^fl/fl^ mice. It showed that the insertions of loxP sites do not interfere with expression of the floxed gene and normal protein production (Fig 2E and 2F). Then, the floxed AQP1 alleles were converted to the deleted AQP1 alleles in a tamoxifen inducible manner by endothelial-specific Cre-mediated excision of exons 2 and 3. The resulting AQP1^fl/fl^; Cdh5-Cre^+^ mice revealed a high efficient EC-specific deletion of the AQP1 in tissues (Figs 3 and 4) with strongly expressed AQP1 such as lung and PM [3,16].

In contrast to global AQP1 knockout mice with impaired survival, lower body weight [16,17], AQP1^fl/fl^; Cdh5-Cre^+^ mice survived at a similar rate as control AQP1^fl/fl^; Cdh5-Cre^-^ mice during the period of tamoxifen treatment until functional analyses, and exhibited similar clinical and biological parameters at baseline as AQP1^fl/fl^; Cdh5-Cre^-^ mice (Table 1). Body weight is known to be an important parameter for interpreting experimental outcomes including peritoneal UF calculation [3, 18]. Therefore, the endothelial cell- and time-specific AQP1 knockout (AQP1^fl/fl^; Cdh5-Cre^+^) mice will provide a more specific experimental model for functional investigation of endothelial AQP1. To our best knowledge, this is the first report on an inducible endothelial cell-specific AQP1 knockout mouse model.

Although the 1-hour 3.86% mini-PET has been validated to be a simple, fast and more practical method for assessing AQP1-related solute-free water transport [23], no specific data have been provided in a mouse PD model yet. In addition, until now no data have showed the association between endothelial AQP1 function and its related clinical relevance for UF in PD. The present study addressed the question, using 1h 3.86% mini-PET in the well-characterised tamoxifen-inducible endothelial cell-specific AQP1 knockout mouse model.

During a 1-hour 3.86% mini-PET, AQP1^fl/fl^; Cdh5-Cre^+^ mice exhibited strongly decreased indices for AQP1-related transcellular water transport (43.0% in net UF, 93.0% in sodium sieving and 57.9% in free water transport) compared to control AQP1^fl/fl^; Cdh5-Cre^-^ mice (Table 2 and Fig 5). The transport rates for urea and glucose were not significantly altered (Table 2).

These data confirm the hypothesis that deletion of endothelial AQP1 significantly decreases water transport during the 3.86% mini-PET, without significant changes in small solute transport parameters. It has also been suggested that the deletion of exons 2 and 3 in AQP1 gene may putatively destroy the symmetrical membrane-spanning “hour-glass” structure of mouse AQP1 protein, which has a great functional impact on the resulting AQP1 channel protein [26].

To demonstrate the UF capacity of AQP1, Ni *et al*. characterised water and solute transport in global AQP1 knockout mice (AQP1 ^-/-^ mice) during a 2-hour unphysiological high glucose (7%) dialysate dwell [3]. In comparison with WT littermates, AQP1^-/-^ mice had no sodium sieving and an about 50% decrease in Net UF and 70% decrease in the initial, solute-free UF. Smit *et al*. quantified water transport in patients during the 3.86% mini-PET [25]. Compared to normal UF patients, UF failure patients showed a 42% decrease in Net UF and a 56% decrease in FWT at the end of the 3.86% mini-PET.

Our data provide the first direct evidence for the crucial role of AQP1 expressed in endothelium during 3.86% mini-PET and thereby validate not only essential predictions of the three-pore model, but also the essential role of endothelial AQP1 in ultrafiltration and free water transport during peritoneal dialysis.

Modulation of endothelial AQP1 might provide a promising therapeutic target for preventing ultrafiltration failure in peritoneal dialysis. These findings may also contribute to an improved understanding of AQP1-endothelial biology, such as angiogenesis, wound healing, tumour spread and organ regeneration.

## Material and Methods

### Generation of Inducible Endothelial Cell-specific AQP1 Knockout Mice

The Cre/loxP system was employed to generate the conditional knockout mice. A targeting vector that encompasses 17.2 kb of the AQP1 locus and a loxP / FRT site–flanked pro- and eukaryotic neomycin selection cassette (Neo-gb2-PGK) was designed (Gene Bridges GmbH, INF 584, Heidelberg, Germany) (Fig 1A). The PGK-gb2-neo template encodes the neomycin/kanamycin resistance gene which combines a prokaryotic promoter (gb2) for expression of kanamycin resistance in E.coli with a eukaryotic promoter (PGK) for expression of neomycin resistance in mammalian cells. Embryonic stem cells (ES cells, 129/Ola) were transfected with the linearised targeting construct, and subjected to G418 selection (300 μg / ml). The surviving clones were screened for homologous recombination by long-range PCR with LATaq DNA polymerase (TaKaRa, Otsu, Japan) and the PCR primers are listed in Table 3.

**Table 3:** Primer sequences and amplification sizes.

Primer (5’-3’ sequence)	PCR amplification sizes
**P1F GGGCTAGGTGCCTTAGTGAATTCCT, P1R TTTGCTCCTTCGCTTTCTGGGCTCA**	**fl-neo, 9190bp; WT, no product**
**P2F CTAAAGCGCATGCTCCAGACT, P2R GCGAGTAAAAGCATTTTGCC**	**fl-neo, 5341bp; WT, no product**
**Probe 3F CAAATTCTTCCCCAGGATCA, Probe 3R TGACCAACGAAGGTATGCTCT**	**415bp**
**Probe 5F TGACACCTCACCATCACACTT, Probe 5R CCTCTACCTTGGATGACCGTA**	**339bp**
**P3F ACCAAAGAGAGGCATCCCTGT, P3R ATTAACCCTCACTAAAGGGCG**	**fl-neo, 3257bp; fl, 1708bp; del, 509bp; WT, no product**
**P4F TCCCCATGTCTGACTCTCACC, P4R TGTTGCTGCTCTAACGTCATC**	**WT,129bp; fl,175bp**
**Cdh5-Cre F GCCTGCATTACGGTCGATGCAACGA, Cdh5-Cre R GTGGCAGATGGCGCGGCAACACCATT**	**720bp**

fl-neo, floxed allele with neo cassette; fl, floxed allele without neo cassette; del, deleted allele; WT, wild type allele

The PCR-positive clones were then confirmed by Southern blot by using 339bp and 415bp fragments of the intron sequence as a 5’ or 3’ probe, respectively. These two fragments were created by PCR from wild type 129/Ola genomic DNA by primers probe 5F/5R and probe 3F/3R (Table 3) and then labelled with alkaline phosphatase using AlkPhos Direct Labelling and Detection system with CDP-star (Amersham). 15 μg of genomic DNAs were digested with Nsi I or Xho I, electrophoresed, transferred to a nylon membrane, hybridised with the labelled probes. Southern blot analysis was also preformed with a neo probe to exclude additional random integrations of the targeting vector DNA.

The correctly targeted ES clones were expanded and injected into C57BL/6 blastocysts (Biotech Lab., Interfacultary Biomedical Faculty, University Heidelberg, Germany) to yield chimeric founder mice. Germline transmission was achieved by mating male chimeric founders with C57BL/6N female mice (The Jackson Laboratory, Maine, USA). The chimeric mice with proven germ line transmission ability (AQP1^fl-neo^) were bred with FLPeR (B6.129S4-Gt (ROSA) 26Sor^tm1(FLP1)Dym^/RainJ) mice (The Jackson Laboratory, Maine, USA) to remove the FRT-flanked neo expression cassette, and then backcrossed to C57BL/6 mice to exclude the FLP allele and produce floxed AQP1 mice (AQP1^fl/fl^). The inducible endothelial cell (EC)-specific AQP1 knockout mouse line was generated by crossing AQP1^fl/fl^ mice with Cdh5 (PAC)-CreERT2 (Cdh5-Cre) mice^22^ to obtain control AQP1^fl/fl^; Cdh5-Cre^-^ littermates and AQP1^fl/fl^; Cdh5-Cre^+^ mice. To inactivate the AQP1 gene, 5-week-old mice (both groups) received intraperitoneal injections or IP injections of tamoxifen (2 mg of tamoxifen, Sigma, T5648; tamoxifen 20mg/ml in Miglyol 812) for 5 consecutive days to induce Cre/loxP mediated gene deletion. All treated mice were not used until two weeks following the last day of tamoxifen treatment.

### Mouse Genotype Analysis by PCR

The locations of all PCR primers used for genotyping are indicated in Fig 1. All PCR primer sequences and their related amplification sizes are listed in Table 3. Mouse genomic DNA samples were prepared from tail tips by following standard protocols.

### Peritoneal Transport Studies

All animal procedures were approved by the University of Heidelberg Animal Care Committee and were performed on mice according to the guidelines of the Institute of Laboratory Animal Science of the University of Heidelberg.

Two weeks after last IP injection of tamoxifen, 8-week-old mice were submitted to a 1h mini-PET with 3.86% glucose-based dialysate (2.0 ml; Baxter Deutschlang GmbH, Munich, Germany) without anaesthesia. Each group contained 6 or 12 mice (n = 6, 4 male and 2 female; n = 12, 8 male and 4 female).

To minimise animal suffering and distress: (i) mice were treated with the shorter dwell time (1 hour) compared to the published data [3]; (ii) at the end of the dwell, mice were anaesthetised with Ketamine (100 mg/kg i.m, Bremer Pharma GMBH, Warburg, Germany) and Xylazine (5 mg/kg i.m, Ecuphar GMBH, Germany), (iii) at the end of the peritoneal transport study (the dialysate samples were recovered from the peritoneal cavity [7] and blood samples were taken from the posterior vena cava), mice were enthanised by cervical dislocation. Peritoneum and lung samples were processed for further investigation, such as immunohistochemistry (IHC) analysis, immunocytochemistry (ICC) analysis and isolation of endothelial cells.

During the 1-hour dwell, mice were monitored every 15 minutes and were enthanised by cervical dislocation if they were in a state of respiratory distress. There were no unexpected deaths and all mice survived during the 1-hour dwell. All parameters from the plasma and dialysate were assessed using Roche/Hitachi cobas c311 in the Medical Research Centre, University Medical Centre Mannheim, Mannheim, Germany. Sodium plasma and dialysate levels were measured with ISE indirect Na, K, CI for Gen.2, glucose levels with Glucose HK in haemolysate Gen.2, and urea with Urea/BUN, respectively. Dialysate creatinine levels were measured with the Jaffe method in the central laboratory of Heidelberg University Hospital, University of Heidelberg, Heidelberg, Germany.

The following calculations were performed [7, 23]:

1. Total net UF (NUF)
  NUF (μl) = (VD60–VD0), where VD0 and VD60 are the volume (μl) of the recovered dialysate at 0 and 60 minutes of the dwell time. V0 was equal to 1.985 ml [an average volume recovered from 6 8-week-old mice (4 males and 2 females), immediately after 2 ml IP injection with 3.86% glucose-based dialysate]
2. Dialysate / plasma sodium concentration (mmol/L) ratio at 0 minute (Na0D/P) and at 60 minutes of the test (Na60D/P).
  Plasma sodium concentration at the start (Na0P) was an average concentration obtained from 6 mice of each group without processing the 3.86% mini-PET.
3. Sodium sieving (%) during the mini-PET
  Sodium sieving(%)=(Na0D/P−Na60D/P)/Na0D/P•100(in%)
4. UF through small pores (UFSP):
  UFSP (μl) = [NaR (mmol/L)*10^6^ / Nap ((mmol/L), where UFSP is UF through small pores, Nap is plasma Na concentration and NaR is sodium removal.
  NaR = (Na60D X VD60) − (Na0D X VD0), where Na60D and VD60 are Na concentration (mmol/L) and volume in the effluent (μl) at 60 minutes of the test. Na0D and VD0 are the Na concentration (mmol/L) and volume (μl) of infused at 0 h of dwell time.
5. Free water transport (FWT):
  FWT(μl)=NUF(μl)-UFSP(μl)
6. Glucose D60/D0 (the dialysate glucose at 60 minutes versus the dialysate glucose at time zero) and Urea 60 D/P (dialysate/plasma urea concentration ratio at the end of the peritoneal equilibration tests.)

According to the established three-pore model of PD, UF occurs either through capillary small pores and accompanied by a solute (e.g. sodium, urea), or transcellular fluid transport that is selective for water only and occurs through specialised AQP1 water channels [22]. Therefore, free water transport (FWT) can be calculated with the La Milia method [23] by using sodium concentration values in dialysate and plasma, as well as NUF.

It has been known that, during the first hour of a hypertonic dwell, free water transport is maximal (since the glucose dialysate concentration is at its peak) and that the diffusive transport of sodium from blood to dialysate is very low (sodium concentration is close to its equilibrium across the peritoneal membrane and thus its gradient for diffusion is very low) [23]. In these conditions, nearly all the sodium transport is due to convection through small pores and thus the ratio of sodium removal (NaR) to plasma water sodium concentration gives the value of UF through the small pores (UFSP). It is then easily possible to calculate FWT by subtracting UFSP from total NUF.

Because the 3.86% mini-PET of La Milia *et al*. only provides more accurate information on free water transport when a correction for sodium diffusion is made [24], a correction for all dialysate sodium (NaD) is made with the mass transfer area coefficient of creatinine as described by Westra *et al*. [28].

NUF, FWT and sodium sieving were used as indexes for transcellular water transport by AQP channels, and glucose D60/D0 and urea D60/D0 ratio were measured to evaluate the peritoneal transport for small solutes [23].

### Immunohistochemistry

The visceral peritoneum (VP) was removed and fixed in 4% paraformaldehyde or zinc for 24 h, and then embedded in paraffin, sectioned (4 μm). Following standard steps [deparaffinising and rehydrating the slides and antigen retrieval (citrate buffer method, pH 6)], paraformaldehyde-fixed VP slides were incubated with 1:100 rabbit anti-AQP1 (Biozol Diagnostica GmbH, Eching, Germany) for 1 h, Histofine Simple stain AP (Multi) Anti rabbit (Nichirei Bioscience Inc, Tokyo, Japan) for 30min, Alkaline Phosphatase-conjugated streptavidin (AP-Label) (Biogenex, Fremont, CA, USA) for 20 min, Hemalum (Carl Roth, Karlsruhe, Germany) for 1 min, and finally detected with Fast-Red.

Zinc-fixed VP slides were washed three times in dH_2_O and blocked (7% BSA, 10% goat serum, 0.2% Triton X-100 in PBS) for 30 min, and then incubated with 1:100 rabbit anti-AQP1 (Biozol Diagnostica GmbH, Eching, Germany) and 1:100 Hamster anti-CD31 (Millipore, Merck Chemicals GmbH, Darmstadt, Germany) overnight at 4°C. Following those steps, the slides were washed three times with 0.2% Triton X-100 in PBS and incubated with secondary antibody [1:200 Cy3-conjugated donkey anti-rabbit IgG (Dianova, Hamburg, Germany); 1:200 Alexa Flour 488 (Dianova, Hamburg, Germany) goat anti-Hamster] and Hoechst 33342 1:1000 for 45 min at room temperature.

To evaluate the microvascular endothelial area or endothelial AQP1 area in mouse VP immunostained for CD31 and AQP1, VP slides from AQP1^fl/fl^; Cdh5-Cre^-^ mice were applied with the same excitation, gain and exposure settings as those of AQP1^fl/fl^; Cdh5-Cre^+^ mice. Processed images were imported to ImageJ where the intensity threshold was set automatically and analysed for units of positive pixel area. The relative endothelial area (the ratio between the CD31-stained area and the entire VP area, in %), or the relative endothelial AQP1 area (the ratio between the AQP1-stained area and the CD31-stained area, in %) was measured. All slides were masked to prevent bias and imaged at 20× magnification. No less than three fields of view (FOV) were obtained per slide, no less than 10–15 FOV were obtained per animal and five animals were evaluated.

### Primary Culture of Endothelial Cells

ECs isolated from lung [29] or VP [30] were cultured from 8-week-old mice by a modified method. Briefly, the lung or VP were minced and digested in collagenase A solution (1 mg/ml, Roche Diagnostics GmbH, Mannheim, Germany) at 37°C for ~60 minutes. The cellular digest was filtered through sterile 40 μm nylon mesh and centrifuged at 800 g for 10 minutes. The cell suspension in 10% FCS–DMEM (Dulbecco's modified Eagle's medium containing 10% foetal calf serum) was incubated with Dynabeads (M-450, sheep-anti-rat IgG, Dynal, Life Technologies, CA, USA) for 30 minutes at 4°C. The bead-bound cells were recovered, washed with 10% FCS–DMEM and FCS-free DMEM, and then digested for 5 to 10 minutes at 37°C with trypsin/EDTA (GIBCO, Life Technologies, CA, USA) to release the beads. The bead-free cells were centrifuged in 10% FCS–DMEM and then resuspended in EC culture medium.

### Immunocytochemistry

ECs cultured on glass coverslips were rinsed with phosphate-buffered saline (PBS) and fixed in 3% paraformaldehyde (PFA) for 5 min. Then cells were incubated with 0.1% Triton X-100 for 1 min. After being washed with PBS, the cells were incubated with primary antibodies (1:50 rabbit anti-AQP1, Biozol Diagnostica GmbH, Eching, Germany; 1:50 Hamster anti-mouse CD31, Millipore Merck Chemicals GmbH, Darmstadt, Germany) in blocking solution (2% FCS, 0.2% fish gelatine, 2% BSA in PBS) for 2 hours at room temperature. The cells were then washed with PBS (three times, 5 min each), and incubated for 45 min at room temperature with secondary antibodies (1:200 Cy3-conjugated donkey anti-rabbit IgG, Dianova, Hamburg, Germany; 1:200 goat anti-Hamster AlexaFlour 488, Dianova, Hamburg, Germany) and 1:1000 Hoechst 33342. Next, the cells were washed three times with PBS, for 5 min each time. Finally, the cells were washed twice in dH_2_O for 3 minutes and mounted for microscopic visualisation.

### Image Acquisition

For the immunoassay, images for cells and tissue slides were acquired using the Nikon Eclipse 80i upright automated microscope with a Nikon Plan Fluor 20x NA 0.50 objective (Nikon GmbH, Düsseldorf, Germany). Image acquisition and processing were controlled by NIS Elements BR 3.0 software. The images were visualised and processed with Image J.

### Western Blot

Tissue or EC samples were homogenised in lysis buffer. Approximately 40μg of tissue protein from VP or 10μg EC protein from lung was separated by 12% SDS-PAGE, transferred to a PVDF Membrane (WesternBreeze Chromogenic Western Blot, Invitrogen, Life Technologies, CA, USA), and incubated with 1:500 rabbit anti-AQP1 (Biozol, Hamburg, Germany) overnight (4°C). Samples were treated with alkaline phosphatase-conjugated anti-rabbit secondary antibody, and immunoreactivity was detected by using the WesternBreeze® Chromogenic Kit according to the manufacturer’s specifications (WesternBreeze Chromogenic Western Blot, Invitrogen, Life Technologies, CA, USA). The intensity of the resulting bands was quantified by densitometry using Image J.

### Statistical Analyses

The statistical data were presented as the means ± S.E. Statistical significance was evaluated with the Student’s t-test for pairwise comparison of data. Differences were considered significant when a p value was < 0.05. In the figures, significance levels are shown as asterisks (“*” for p < 0.05, “**” for p < 0.01, “***” for p < 0.001).
